# Insertable Biomaterial-Based Multianalyte Barcode Sensor toward Continuous Monitoring of Glucose and Oxygen

**DOI:** 10.1021/acssensors.4c01926

**Published:** 2024-11-04

**Authors:** Ridhi Pradhan, David Chimene, Brian S. Ko, Artem Goncharov, Aydogan Ozcan, Michael J. McShane

**Affiliations:** † Department of Biomedical Engineering, Texas A&M University, College Station, Texas 77843, United States; ‡ Electrical & Computer Engineering Department, University of California, Los Angeles, California 90095, United States; § Bioengineering Department, University of California, Los Angeles, California 90095, United States; ∥ California NanoSystems Institute (CNSI), University of California, Los Angeles, California 90095, United States; ⊥ Department of Surgery, University of California, Los Angeles, California 90095, United States; # Department of Materials Science and Engineering, Texas A&M University, College Station, Texas 77843, United States

**Keywords:** continuous glucose monitoring, oxygen monitoring, barcode, hydrogel, insertable, multianalyte, biocompatible, optical sensor

## Abstract

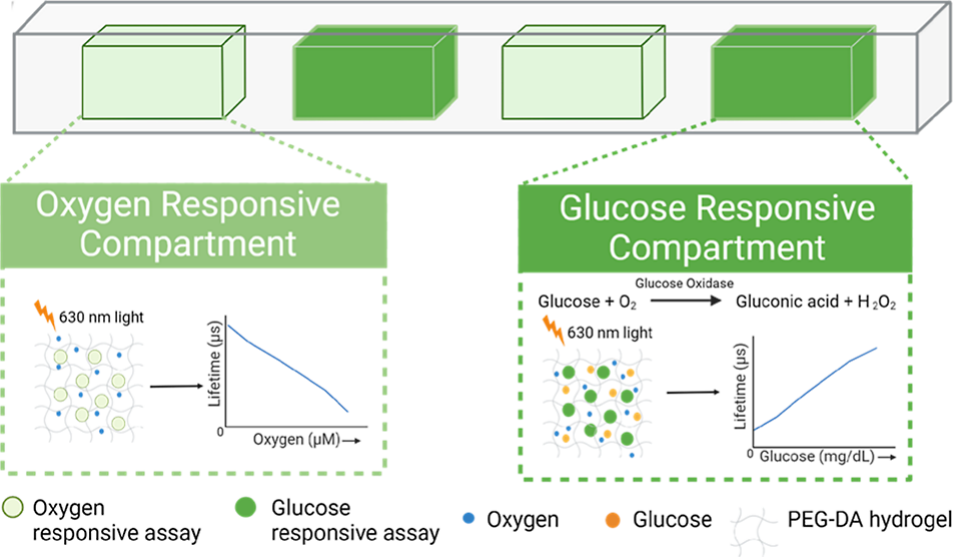

Chronic diseases, including diabetes, cardiovascular diseases, and microvascular complications, contribute significantly to global morbidity and mortality. Current monitoring tools such as glucometers and continuous glucose monitors only measure one analyte; multiplexing technologies offer a promising approach for monitoring multiple biomarkers, enabling the management of comorbidities and providing more comprehensive disease insights. In this work, we describe a miniaturized optical “barcode” sensor with high biocompatibility for the continuous monitoring of glucose and oxygen. This enzymatic sensor relies on oxygen consumption in proportion to local glucose levels and the phosphorescence reporting of tissue oxygen with a lifetime-based probe. The sensor was specifically designed to operate in a tissue environment with low levels of dissolved oxygen. The barcode sensor consists of a poly(ethylene glycol) diacrylate (PEGDA) hydrogel with four discrete compartments separately filled with glucose- or oxygen-sensing phosphorescent microparticles. We evaluated the response of the barcode hydrogels to fluctuating glucose levels over the physiological range under low oxygen conditions, demonstrating the controlled tuning of dynamic range and sensitivity. Moreover, the barcode sensor exhibited remarkable storage stability over 12 weeks, along with full reversibility and excellent reproducibility (∼6% variability in the phosphorescence lifetime) over nearly 50 devices. Electron beam sterilization had a negligible effect on the glucose response of the barcode sensors. Furthermore, our investigation revealed minimal phosphorescence lifetime changes in oxygen compartments while exhibiting increased lifetime in glucose-responsive compartments when subjected to alternating glucose concentrations (0 and 200 mg/dL), showcasing the sensor’s multianalyte sensing capabilities without crosstalk between compartments. Additionally, the evaluation of chronic tissue response to sensors inserted in pigs revealed the appropriate biocompatibility of the barcodes as well as excellent material stability over many months. These findings support further development of similar technologies for introducing optical assays for multiple biomarkers that can provide continuous or on-demand feedback to individuals to manage chronic conditions.

Chronic diseases are long-lasting health-related problems affecting the quality of life of many people and causing morbidity and mortality worldwide. Chronic disease causes nearly 41 million deaths every year, equivalent to 74% of all deaths globally.^1^ According to the World Health Organization (WHO), chronic diseases such as cardiovascular disease (CVD), cancer, stroke, chronic obstructive pulmonary disease, and diabetes are the leading causes of death and disability in the United States. In many cases, patients experience comorbidities, such as diabetes and chronic renal failure, or diabetes and microvascular diseases.^2^

Diabetes is the most common endocrine disorder and is a leading cause of mortality worldwide. According to the WHO, in 2019, diabetes mellitus was responsible for 1.5 million deaths. Additionally, 460,000 kidney disease deaths and 20% of cardiovascular deaths were caused by diabetes.^1^ Continuous glucose monitoring (CGM) provides real-time glycemic monitoring to improve diabetes management and health outcomes. In addition to glucose monitoring, regional tissue oxygen is a key interest in detecting and managing microvascular disease in diabetic patients.^3,4^ The reduced regional oxygen levels in diabetic patients can contribute to the progression of complications associated with microvascular diseases including nephropathy, retinopathy, and albuminuria. Consequently, measuring regional tissue oxygen emerges as a crucial aspect in identifying microvascular diseases in diabetic patients.^4^ Additionally, the surrounding uneven oxygen distribution within the tissues can also interfere with CGMs, leading to inaccuracies in the readings of the glucose concentrations in the body, and physiological oxygen concentrations can fluctuate due to inflammation, especially after implantation, hindering the stable response of the CGM device.^5−7^ Hence, employing a multianalyte sensor capable of assessing tissue oxygen levels, alongside CGM, could serve as an invaluable diagnostic tool for preventing microvascular diseases, effectively managing diabetes in affected individuals, and maintaining the accuracy of CGMs even when the oxygen level changes at an implantation site.

The commercially available electrochemical CGMs such as Abbott Libre, Dexcom, and Medtronic Guardian that are partially implanted pose constant sensor tissue friction, shortening the longevity of these sensors to 3–14 days.^8,9^ Alternatively, optical measurements including spectroscopy (NIR, Raman) and fluorescence have shown high potential in long-term implantable continuous glucose sensing.^10,11^ Eversense, an optical-based CGM (measuring 3.5 mm × 18.3 mm), has been developed by Senseonics, which is implanted into the subcutaneous tissue of the upper arm through a surgical procedure using local anesthesia. Currently, only this sensor presents a longer lifespan (180 days) compared to electrochemical CGM sensors. Nevertheless, challenges related to invasiveness and biocompatibility (foreign body reaction, FBR) persist. FBR hampers metabolite diffusion and degrades optical chemistry, ultimately causing inaccuracies in the sensor’s readings. The intensity and nature of the inflammation cascade are intricately linked to the initial inflammatory response, particularly the adsorption of biomolecules, as well as the characteristics of the foreign body such as size, shape, and physical and chemical properties.^12,13^ Therefore, miniaturizing implantable CGMs is crucial to ensure less invasive insertion and to minimize the risk of potential immune reactions against the sensor.

A continuous monitoring system offers prolonged tracking of physiological parameters and plays a critical role in chronic disease management. Continuous monitoring devices can detect abrupt or unforeseen fluctuations between discrete measurement times, enabling early intervention, personalized care, and reduced hospitalizations by identifying changes in real time.^8^ The notable success of CGM to improve the therapeutic outcome of patients over the past decades has sparked a growing interest among researchers and clinicians in the continuous sensing of additional biomarkers.^11^ However, in some cases, the existence of comorbidities may only be identified by monitoring the concentration of multiple biomarkers. Additionally, relying solely on the detection of a single biomarker often needs to be revised for clinical diagnosis or monitoring of disease progression. Multiplexing enables the simultaneous detection of multiple analytes, which can significantly improve the management of comorbidities by providing more information about the disease(s) and its/their status.^14,15^ Multianalyte sensors also have the advantage of providing a shorter overall analysis time for multiple analytes within the sample volume, offering a cost-effective and rapid analysis over biosensing of individual analytes.

Some recent papers have reported on insertable optical sensors that do not require implanted electronics as an alternative to electrochemical sensors; these typically rely on phosphorescent oxygen-sensitive luminescent dye embedded into hydrogel matrices.^16−18^ To make these materials responsive to other analytes, oxidoreductase enzymes are also included to enable the measurement of, for example, glucose, oxygen, and other analytes.^19−22^ In these systems, a luminescent dye/phosphor is collisionally quenched by oxygen, reducing its phosphorescence lifetime; thus, the lifetime is proportional to the local oxygen concentration. The biosensing assays indirectly respond to a target analyte by depleting local oxygen mediated by specific oxidoreductase enzymes. This depletion alters the phosphorescence lifetime in proportion to the analyte concentration: the reduction of oxygen in the system increases the phosphorescence lifetime, which is then used to estimate the concentration of the target analyte using a known relationship determined by precalibration.

We have recently reported a multicompartment poly(ethylene) glycol diacrylate (PEG-DA) miniaturized implantable hydrogel called the “barcode”, which has dimensions comparable to a rice grain and can be easily inserted into subcutaneous tissue using a 16-gauge needle.^23^ These barcode sensors encapsulated discrete assays based on phosphorescent Pd(II) metalloporphyrin dyes for continuous monitoring of glucose and oxygen with minimal crosstalk. The glucose-sensing and oxygen-sensing microparticles consisted of two different porphyrin dyes. The glucose-responsive microparticles consisted of Pd (II) *meso*-tetra (sulfophenyl) tetrabenzoporphyrin sodium salt (HULK) and the oxygen-sensing microparticles consisted of [Pd-*meso*-tetra(4-carboxyphenyl) porphyrin (PdP). The HULK has a red excitation wavelength of 630 nm, which falls within the optical window of human skin. However, PdP has an excitation wavelength near 530 nm, which can cause difficulty in excitation in vivo due to the absorption and scattering of light by skin tissue. In addition, it is notable that those initial proof-of-concept experiments for these barcode sensors were performed at oxygen levels much higher than those of interstitial tissue oxygen.^10^ Hence, these sensors quickly saturated at relatively low analyte concentrations when used in typical low oxygen concentrations found in tissue (∼30–40 μM).^4^ Moreover, several critical factors that are essential for ensuring stable sensor performance prior to in vivo studies, such as sterilization, reproducibility, storage conditions, enzyme stability, and in vivo biocompatibility, were not previously evaluated.

This work aimed to advance the barcode sensor for improved sensitivity and stability by optimizing the design for performance at lower interstitial oxygen conditions.^24^ To accomplish this, we replaced the HULK porphyrin dye with oxygen-sensitive ethyl cellulose (EC) nanoparticles (ECNPs) encapsulating a palladium(II) *meso*-tetra(4-carboxyphenyl)tetrabenzo-porphyrin) dye (PdBP). ECNPs have proved to be more sensitive compared to the free form of the dye.^25^ Additionally, to tune the sensitivity and dynamic range of these sensors under lower oxygen conditions, the glucose-sensing microparticles were further modified with cross-linked diffusion-limiting polyelectrolyte coatings to control the glucose permeation more precisely into the sensing domains. Furthermore, the stability, reproducibility, effects of sterilization, reversibility, and biocompatibility of the barcode sensors were assessed.

## Experimental Section

### Reagents and Materials

Alginate (Cat no. A2158, 75–100 kDa), EC (Cat no. 200697, 48% ethoxy), TRIS base (Cat no. T1503), [2-(methacryloyloxy) ethyl]trimethylammonium chloride (TMA, Cat. no. 408107, 80% in water), catalase (CAT, from bovine liver, Cat. no. C9322), calcium chloride (Cat no. 22350), pluronic F 68 (PF68, Cat. no. P1300), poly(allylamine hydrochloride) (PAH, Cat no.71550-12-4, 17.5 kDa), poly(sodium-4-styrenesulfonate) (PSS, Cat no. 25704-18-1, 70 kDa), 2,2- dimethoxy-2-phenyl acetophenone (C_6_H_5_COC(OCH3)2C_6_H_5_, >99%), 1-vinyl-2-pyrrolidinone (C_6_H_9_NO, >99%), and tetrahydrofuran (THF, Cat no. 401757) were purchased from Sigma-Aldrich, Inc., St. Louis, MO, USA. PdBP (Cat no. T13343) was purchased from Frontier Specialty Chemicals, Logan, UT, USA. Iso-octane (Cat no. 94701) was purchased from Avantor performance materials, LLC, Randor, PA, USA. Glucose oxidase (GOx, Cat no. 9001-37-0, Activity—76.8 unit/mg) from *Aspergillus niger* was purchased from Tokyo Chemical Industries Co. (Tokyo, Japan). PEGDA (average *M*_w_ ∼ 3.4 kDa) was purchased from Alfa Aesar (Haverhill, MA, USA).

### Synthesis of Nanoparticles Containing Oxygen-Sensitive Phosphors

The oxygen-sensitive nanoparticles were fabricated using the previously reported nanoemulsion method.^25^ Briefly, 100 mg of EC was dissolved in 5 mL of THF overnight using a magnetic stirrer. Then, 2 mg of PdBP was dissolved into the above solution of EC and THF using sonication for 30 min. The above THF solution with the dye and polymer was filtered through a 0.2 μm PTFE syringe filter and stored in a vial. In a separate 50 mL centrifuge tube, 100 mg of surfactant (PF 68) was dissolved in 20 mL of nanopore water using sonication and filtered through a 0.2 μm PTFE syringe filter. Next, the surfactant solution was sonicated using a sonication probe, and the THF solution with dye and polymer was slowly injected into the aqueous solution of surfactant within 30 s of the 2 min sonication process. Finally, the suspension of NPs was filtered through a 100 μm nylon filter to remove larger precipitates, and the volume was reduced to 3 mL by using centrifuge filtration. Then, the NP suspension was washed with 15 mL of nanopure water to remove excess surfactant. These NPs were stored in a vial of 16.6 mg/mL in the fridge (4 °C) for further use.

### Synthesis of Sensing Microparticles with Nanofilms

**Figure 1:**
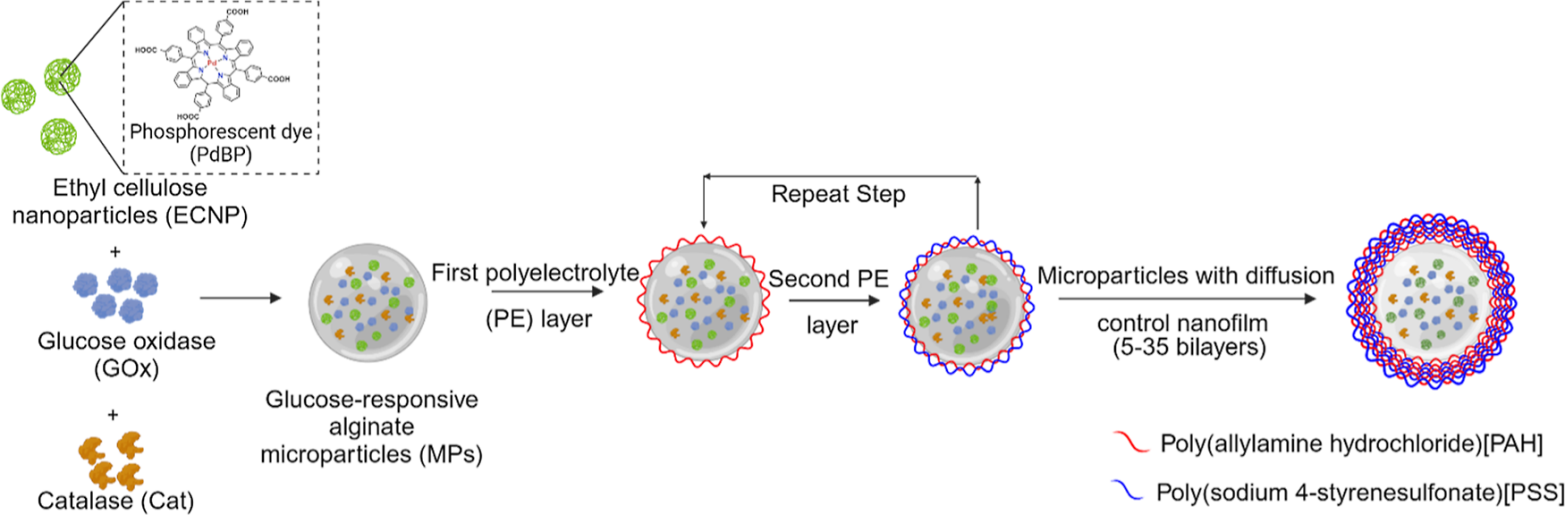
Illustration of alginate microparticles encapsulating oxygen-sensitive EC nanoparticles (ECNPs), GOx, and catalase (Cat) coated with polyelectrolyte multilayer for glucose diffusion control. Multilayer nanofilms were deposited by the layer-by-layer sequential electrostatic adsorption method.

Alginate microparticles encapsulating the oxygen-sensitive ECNPs, GOx, and catalase (Cat) were synthesized by using an emulsion technique. GOx is widely considered the preferred functional material for glucose sensing as it has good sensitivity and selectivity toward glucose detection.^26,27^ A homogeneous mixture of 3.75 mL of 4% w/v aqueous solution of sodium alginate and 1.25 mL (5 mg NPs) of nanoparticle suspension was obtained by nutating the mixture for 30 min. In a separate tube, 58.5 mg of GOx and 54.9 mg of Cat were dissolved in 2.5 mL of 50 mM TRIS buffer (pH 7.2) by gentle nutation. Then, the alginate and enzyme mixtures were mixed to prepare a precursor solution. This mixture was added dropwise and emulsified for 2 min in a solution containing 10.8 mL of isooctane with 322 μL of SPAN 85 using a homogenizer operating at 8000 rpm. Next, 1.5 mL of isooctane with 175 μL of TWEEN 85 was added to the above mixture and stirred with the same speed for 15 s. During the last 50 s of the emulsification, 4 mL of 10 w/v % CaCl_2_ solution was added to allow external gelation of the alginate microparticles. The emulsion was then transferred to a round-bottomed flask and washed with deionized (DI) water two times. Next, the surfaces were gently stirred in a magnetic stirrer for 20 min. The microparticles were centrifuged at 2000*g* for 2 min, and the microparticles were modified by applying ultrathin nanofilms of polyelectrolytes using the electrostatic layer-by-layer (LbL) assembly method. Figure 1 illustrates the fabrication of sensing alginate microparticles bound to polyelectrolyte nanofilms.

The oxygen-sensing microparticles were synthesized by using the same method used for synthesizing the glucose-sensing microparticles, excluding the enzymes.

### Layer-by-Layer Deposition

Polyelectrolyte nanofilms were deposited on the alginate microparticles by dispersing the pallet of the microparticles in 2 mL of PAH (pH 8) followed by centrifugation, and the supernatant was discarded. Then the pallet was resuspended in PAH wash solution (10 mM TRIS buffer with pH 8) and centrifuged, and the supernatant was discarded. The same procedure was followed for PSS (pH 7.2) and PSS wash solution (10 mM TRIS buffer at pH 7.2). This created one bilayer of polyelectrolytes PAH and PSS. The microparticles for oxygen sensing consisted of 5 bilayers. The microparticles for glucose sensing consisted of a total of 5, 10, 15, 20, 25, 30, and 35 bilayers. Further, covalent cross-linking of the amine group of PAH of the glucose-sensing microparticles was also performed by mixing 8.8 mg of nanofilm-coated microparticles with 2 mL of 0.1 M glutaraldehyde and stirring for 30 min. The microparticles were then suspended in a 10 mM TRIS buffer and stored at 4 °C for future use.

The assembly of polyelectrolyte layers on alginate microspheres was monitored by electrophoretic mobility measurements (zeta potential, Malvern Zetasizer Nano ZS). Cellometer Mini (Nexcelom) was used to determine the size of alginate microparticles after LbL depositions. The stock microparticle solution was diluted 1:20 in TRIS buffer. Then, 20 μL of the diluted solution was dispensed onto the slide for analysis.

### Fabrication of the Barcode Hydrogel Sensor

To create a discrete compartment barcode sensor, a previously established soft lithography process was used.^23^ A PDMS top and bottom master mold were fabricated by replica molding from a 3D-printed master mold. The PDMS, the precursor, and the curing agent were mixed at a ratio of 10:1, poured into the printed master mold, and cured at 60 °C under vacuum for 2 h. The hydrogel solution was prepared by mixing 20% (w/v) PEGDA and 2% (v/v) of the photoinitiator solution. The hydrogel precursor solution was dispensed into the bottom master mold, and the top master mold was aligned. The hydrogel was cross-linked under a UV lamp (360 nm, 10–15 mW cm^–2^) by 5 min exposure. The hydrogel case was then peeled from the PDMS mold and rinsed with DI water. PEGDA has been used widely for fabricating hydrogels in various biomedical applications such as controlled drug delivery, implants, biosensors, and tissue scaffolds. PEG hydrogels are inert, biocompatible, and nonimmunogenic and have favorable cell viability, and its mechanical properties can easily be tailored.^28−32^

**Figure 2:**
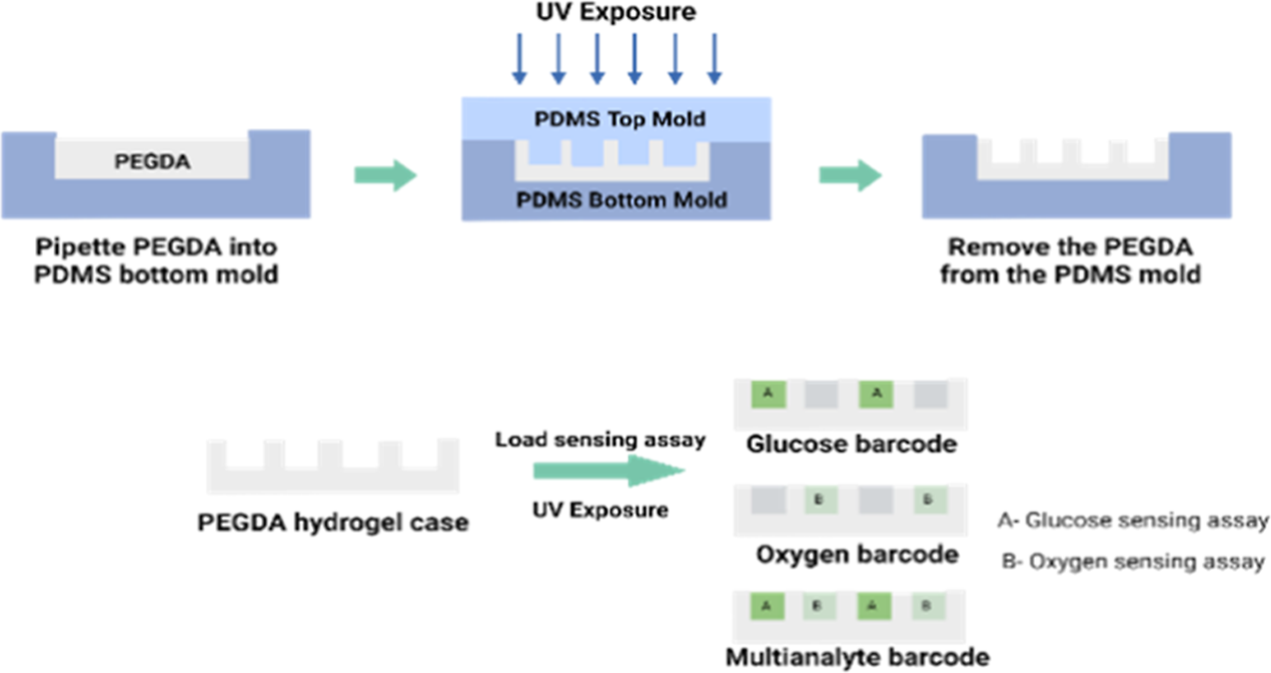
Illustration of the fabrication process of barcode hydrogel sensors using soft lithography.

The sensing assay (8.8 mg/90 μL) was mixed with the hydrogel precursor in a ratio of 3:1. For the glucose sensor termed the “glucose barcode”, glucose-sensing microparticles were mixed with the hydrogel precursor, and the “oxygen barcode” only had oxygen-sensing microparticles. Then, 0.64 μL of the mixture was pipetted into 2 compartments and polymerized under UV for 5 min. For the multianalyte barcode containing glucose- and oxygen-sensing assay, two alternate compartments were filled with a mixture of hydrogel precursor and glucose-sensing microparticles, and the remaining compartments were each filled with a mixture of hydrogel precursor and oxygen-sensing microparticles. After polymerization, the barcode sensors were rinsed in DI water and stored in a TRIS buffer at 4 °C. The biocompatibility of the sensing assay (alginate microparticles) was previously tested through cell viability assays demonstrating negligible cell toxicity.^25^Figure 2 illustrates the fabrication process of barcode hydrogel sensors.

### Characterization of Oxygen and Glucose Response

The barcode hydrogels (*n* = 4) were immobilized in a previously described custom-built flow cell consisting of an acrylic sheet that holds up to four samples inside and attaches optical readers from outside.^33^ Oxygen challenges were performed by exposing the sensors to varying dissolved oxygen concentrations inside an incubator at 37 °C. The dissolved oxygen concentration of 0–257.9 μM was achieved by mixing air and nitrogen in a defined ratio between 0 and 21% using a digitally controlled mass flow controller (MKS Instruments PR 4000B). The glucose challenges were performed by exposing the sensors to varying physiologically relevant glucose concentrations (0–400 mg/dL) in an incubator (37 °C) while holding oxygen at a fixed concentration of ∼40 μM, representative of expected tissue levels. The glucose concentration was modulated using peristaltic pumps (Masterflex, 7550-50) by mixing feeds drawn from reservoirs containing 0 and 400 mg/dL of glucose stock solution in 10 mM TRIS with 10 mM CaCl_2_. The pump speeds were controlled by a custom LABVIEW program (Figure S1) to precisely mix the solutions to achieve glucose levels over a full range of concentrations. The oxygen concentration was regulated using a vacuum degassing chamber (9000-1118) and vacuum pump (9000-1472-Systec).^34^ All lifetime readings (Figure S4) were recorded using a custom multichannel time-domain phosphorescence lifetime measurement system with 630 nm excitation and 800 nm emission.^33^

The multiplexing capabilities of the barcode hydrogel sensors were further tested using a custom phosphorescence lifetime imager (PLI), which can spatially resolve responses from different compartments of the barcode devices.^35^ The PLI reader acquired time-lapse phosphorescence images of decaying sensor emissions, which were then processed to obtain phosphorescence intensity and lifetime images of the entire field of view. The lifetime responses of the hydrogel sensor compartments were further calculated by averaging pixels within rectangular masks overlaid with each compartment, yielding four signals per device (two glucose-sensitive compartments and two oxygen-sensitive compartments). Final lifetime values for each analyte were obtained by averaging responses from alike compartments.^35^

The limit of detection (LOD) was estimated from a linear regression model by calculating the glucose concentration corresponding to the phosphorescence lifetime imager at 0 mg/dL glucose plus 3 times the standard deviation (SD) of the lifetime signal at that analyte concentration. Similarly, the maximum differentiable glucose concentration (MDGC) was estimated by calculating the glucose concentration corresponding to the phosphorescence lifetime at 400 mg/dL glucose plus 3 times the SD of the lifetime signal at that analyte concentration. The dynamic range was calculated as R = MDGC × LOD, and the sensitivity was calculated by dividing the difference in phosphorescence lifetime values at LOD and MDGC by the dynamic range.

### Assessment of Storage and Operational/Enzyme Stability

Barcodes were incubated under two different conditions to evaluate their long-term stability under relevant circumstances. For evaluating the storage stability, samples were stored in a 10 mM TRIS buffer containing no glucose at 4 °C. The lifetime responses from these sensors were assessed at the beginning, 4, and 12 weeks. In the second condition, the operational/enzyme stability of the barcode sensors was evaluated by incubating samples in physiological solutions of 10 mM phosphate-buffered saline (PBS) containing 100 mg/dL glucose at 37 °C for 8 weeks. Over the duration of storage, the buffer containing the samples was tightly sealed, stirred, and changed every 4 days. The enzymatic activity of GOx was monitored using a colorimetric assay that relies on the oxidation of *o*-dianisidine within a peroxidase-coupled system. To conduct the assay, a reaction cocktail, consisting of a cocktail of glucose, *o*-dianisidine, and horseradish peroxidase in sodium acetate, was prepared according to an established protocol.^36^ The sensors were placed into a 96-well plate, 200 μL of the reaction cocktail was dispensed into each well, and absorbance at 492 nm was monitored in 1 min kinetic interval cycles for 30 min using a plate reader (BioTek Cytation 5). The experiment was repeated for the samples after fabrication and biweekly upon storage under physiological conditions. The change in absorbance was plotted over time, and the slopes of the linear portion of the curves were used to determine the comparative apparent activity among the samples at different time points.

### In Vivo Insertion

To better understand the long-term biocompatibility and performance of the barcode samples under real-world conditions, barcode sensors were inserted under the skin of two female pigs. Female piglets were used for ease of handling so that the pigs could continue to live together after puberty, which occurs roughly at 6 months old. As a production breed, Yorkshire cross pigs are known for robust health and gregarious nature, making them well suited to an extended study. The pigs lived together in semi-enclosed housing, with access to toys and enrichment, including a wading pool and fans, and heat lamps and hay beds as appropriate for the season. This animal study was IACUC-approved under AUP# IACUC 2021-0066 Reference Number: 137661.

The barcode sensors were inserted into the pigs at different time points: at 3 months old in Pig 1 and at 7.5 months old in Pig 2. Accordingly, the barcode sensors were evaluated over 7 months in Pig 1 and 3 months in Pig 2. During insertion, pigs were anesthetized using isoflurane to minimize stress and intubated for safety. The animals were placed on a heating pad and under a blanket where possible to maintain body temperature. Insertion sites were trimmed and shaved and then cleaned with detergent and isopropyl alcohol. A small marking was tattooed around each insertion site because the implants cannot be visually identified otherwise. The sensors were loaded into 16-gauge needles and inserted subcutaneously to a target depth of 2 mm. A steel dowel rod was used to hold the sensor in place as the needle and dowel rod were withdrawn. After implantation, the pigs were kept under observation until the anesthesia had worn off and then returned to the main pen.

After the experiment, the pigs were humanely euthanized by veterinary staff. The fresh hides containing the sensors were then skinned off and stored in 10% neutral buffered formalin for at least 24 h. The hides were then sectioned off, using the tattoos and lifetime readings to precisely locate each sensor. Roughly one square inch of hide surrounding the strongest sensor reading was then removed, serially sectioned, paraffin-embedded, and stained with hematoxylin and eosin (H&E). Independent veterinary pathologists then examined the slides for device presence, device state, skin zone, host interface, and healing response.

### Statistical Analysis

All data are expressed as mean ± one SD. One-way analysis of variance tests was performed in Origin Pro for comparison between groups. A *p*-value >0.05 was considered statistically significant.

## Results and Discussion

### Characterization of Alginate Microparticles and Barcode Hydrogels

**Figure 3:**
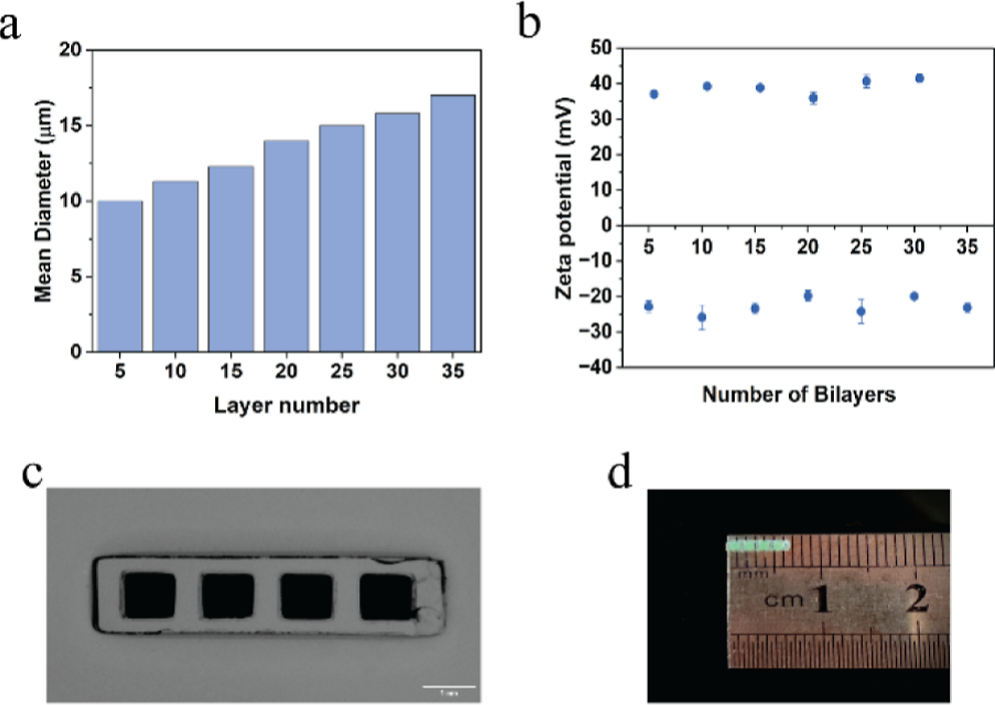
(a) Average diameter of synthesized alginate microparticles bounded with nanofilms of different number of bilayers. (b) ζ-Potential measurements for deposition of polyelectrolyte nanofilms on alginate microparticles. (c) Microscopic image of barcode hydrogel sensor. Scale bar = 1 mm. (d) Image of barcode hydrogel (approximately 6.5 mm long and 1.2 mm wide).

The size and surface charge of alginate microparticles with different LbLs were characterized using the cellometer and zeta potential analyzer, respectively, to ensure proper deposition of polyelectrolyte layers. The diameter of microparticles coated with 5–35 bilayers of PAH/PSS ranged from 10 to 18 μm, respectively (Figure 3a). The size of the microparticles increased slightly with the increments in the polyelectrolyte bilayers. The zeta potential results demonstrated the reversal of the surface charge of the microparticles at each step from −20 mV for PSS to +40 mV for PAH (Figure 3b). The increasing size and reversal charge indicate the deposition of PAH/PSS layers on the alginate microparticles.^37^ The fabricated barcode hydrogel had a one-by-four linear array of hollow cuboid structures with overall dimensions of 6.5 mm in length, 1.2 mm in width, and 1 mm in height. The individual compartments were each 1 mm long, 0.8 mm wide, and 0.8 mm tall, and the compartments were separated by 0.5 mm of hydrogel. The stereotaxic microscopy images of the barcode hydrogel (Figure 3c) demonstrate the filled compartments, which appear darker than the hydrogel casing.

### Barcode Oxygen and Glucose Response

**Figure 4:**
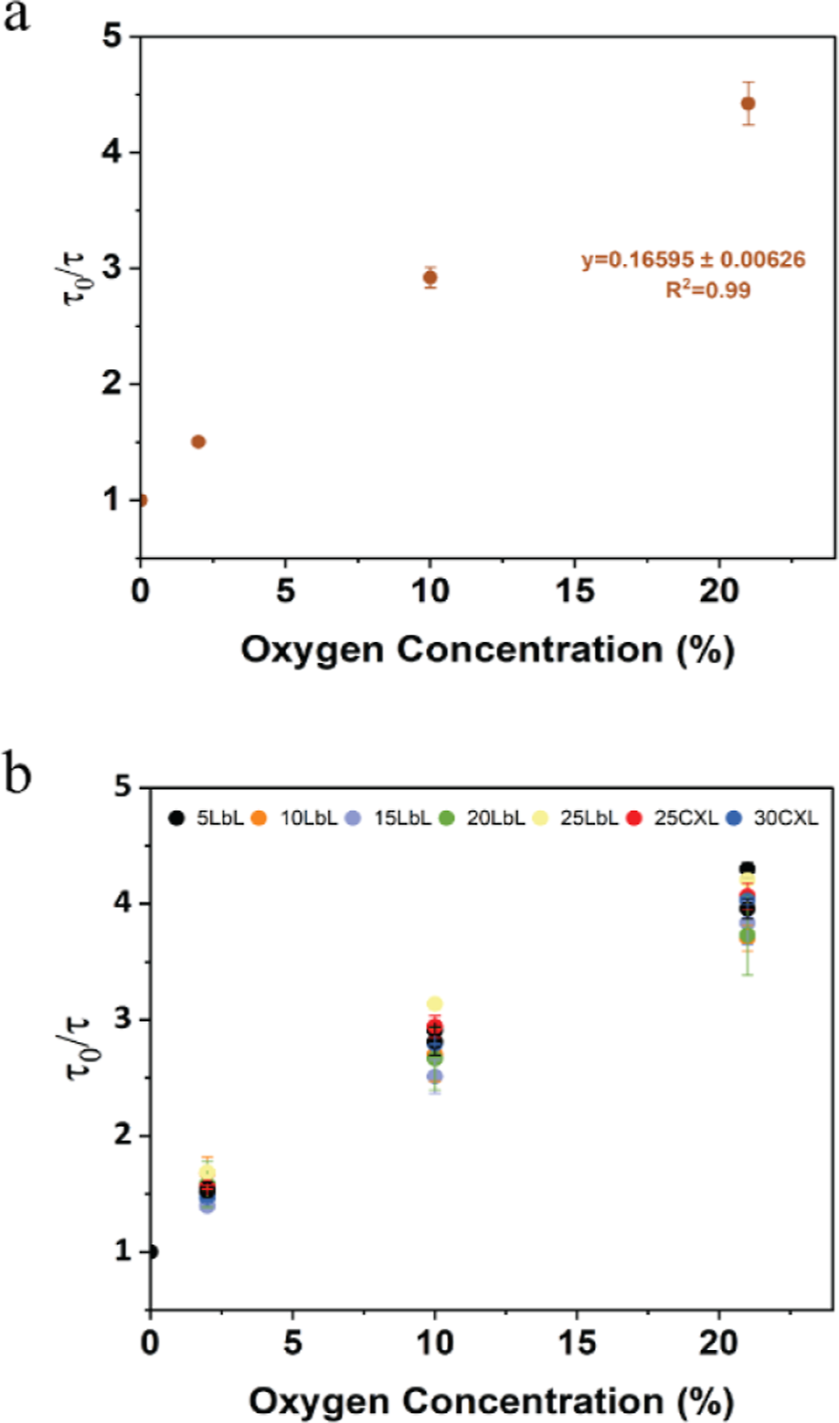
Stern–Volmer plot for (a) oxygen barcode hydrogel sensor and (b) glucose barcodes packed with alginate microparticles bound by varying nanofilm coatings under different oxygen concentrations. Error bars represent the SD from the mean for *N* = 4 different samples.

The lifetime response of the glucose and oxygen barcode sensors to the changes in the oxygen concentrations was measured. The linear Stern–Volmer relationship: τ_0_/τ = 1 + *K*_SV_[O_2_] was used to evaluate the oxygen response. The oxygen-sensitive compartments exhibited a pronounced response to changes in the oxygen concentration from 0% to 21% (0–257.9 μM), as expected (Figure 4a), with a phosphorescence lifetime range of 61–272 μs and a Stern–Volmer constant (*K*_SV_) value of 0.16% O_2_^–1^ (Figure S2). The glucose-sensitive compartments were also evaluated for the response to the changing oxygen concentration, from which it was determined that there was no significant difference in the *K*_SV_ (summarized in Table S1) between these compartments and the oxygen-sensitive compartments; further, there was no significant difference among the particles with different bilayers of nanofilms whether cross-linked or not, indicating that the nanofilms did not substantially affect the kinetics of oxygen diffusion (Figure 4b).

**Figure 5:**
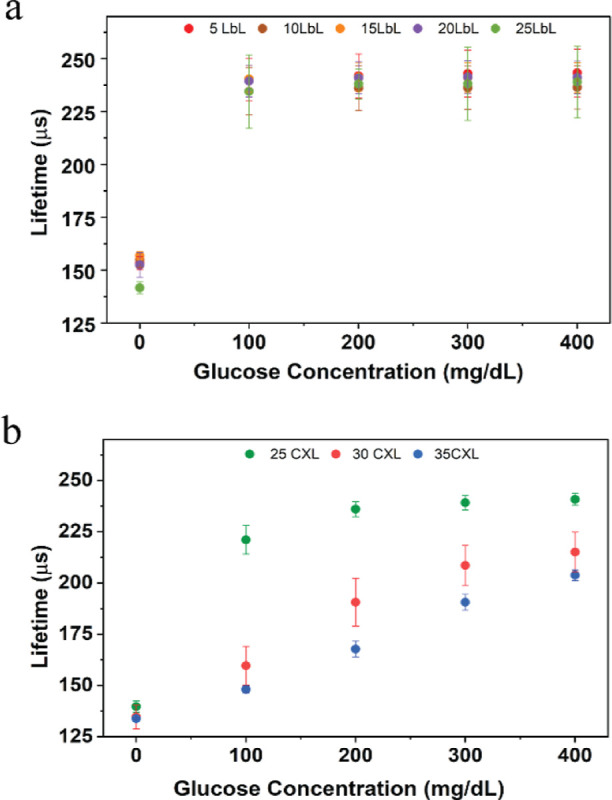
Steady-state lifetime response plots of glucose barcodes (*n* = 4) packed with alginate microparticles with different LbLs, dispersed in barcode hydrogel by challenging the sensors to the physiological glucose concentration (0–400 mg/dL) at 40 μM dissolved oxygen concentration and 37 °C. (a) Sensors consisted of non-cross-linked nanofilm coatings. (b) Sensors consisted of glutaraldehyde cross-linked (CXL) nanofilm coatings. Error bars represent the SD from the mean for *N* = 4 different samples.

The glucose barcode hydrogels were subjected to testing at physiologically relevant glucose concentrations (0–400 mg/dL) under a hypoxic oxygen environment (35–40 μM). When the number of bilayers used to construct the nanofilm coatings was increased from *n* = 5 to *n* = 25, the dynamic range of response to glucose exhibited an increase of ∼145%. The non-cross-linked microparticles with the most bilayers (*n* = 25) yielded a maximum detectable concentration of 133 mg/dL with a sensitivity of 0.37 μs-dL/mg. The expansion of the dynamic range of these sensors is directly related to the increase in the number of bilayers, an effect attributed to a decrease in the flux of glucose into the sensing domains with thicker films, thus decreasing the sensitivity. This result aligns with previous reports on flux-based enzymatic glucose sensors, which showed an increase in the dynamic range and a simultaneous decrease in sensitivity with the decrease of glucose diffusion while maintaining the oxygen diffusion rates.^38^ Despite having 25 bilayers, when tested under hypoxic conditions, the barcode sensor exhibited sensitivity to glucose levels only in the hypoglycemic range (0–100 mg/dL); the lifetime saturated between 225 and 250 μs and failed to further respond to the hyperglycemic levels, as shown in Figures 5a and S3. This suggests that glucose diffusion through the sensing domain remains high, resulting in complete consumption of the limited oxygen even at lower glucose levels. Hence, to further decrease the diffusion of glucose into the sensing domains and extend the concentration dynamic range in hypoxic conditions, the nanofilms were cross-linked using glutaraldehyde. It is known that when the PAH/PSS nanofilms are treated with glutaraldehyde, the amino group of the PAH in the nanofilm readily reacts with the aldehyde group of glutaraldehyde;^38^ this has previously been proven to be an effective method to reduce glucose permeation into the microparticles without hindering the diffusion of oxygen.^20,38^ Cross-linking the 25-bilayer nanofilms increased the dynamic range by ∼16% yielding a maximum detectable concentration of 175 mg/dL. Further increasing the cross-linked nanofilms to 35 bilayers further increased the dynamic range by 84% (Figure 5b). The sensor demonstrated linear response (*R*^2^ > 0.99) within the physiological glucose concentrations of 0–400 mg/dL. The lower range of LOD and MDGC of the sensors was 37 and 321 mg/dL, respectively, with a sensitivity of 0.19 μs-dL/mg. The dynamic range and sensitivity of each sensor type are summarized in Table S1. Hence, considering crucial factors like linear response and sensitivity, MPs with 30 LbLs with glutaraldehyde cross-linked nanofilms were selected for subsequent in vitro analysis.

### Sterilization and Reproducibility

Sterilization is a crucial prerequisite for implantable sensors to ensure their safe and effective use in clinical applications. However, enzymatic sensors can be sensitive to sterilization processes, leading to a loss of sensor activity. Specifically, using heat and toxic gases such as ethylene oxide for sterilization of enzymatic sensors can cause irreversible denaturation of the enzymes.^39^ As a result, radiation-based sterilization has become a widely adopted approach for enzymatic sensors. Here, the barcode sensors were sterilized using electron beam (e-beam) radiation, which offers advantages in terms of affordability and ease of control when compared with γ radiation.

**Figure 6:**
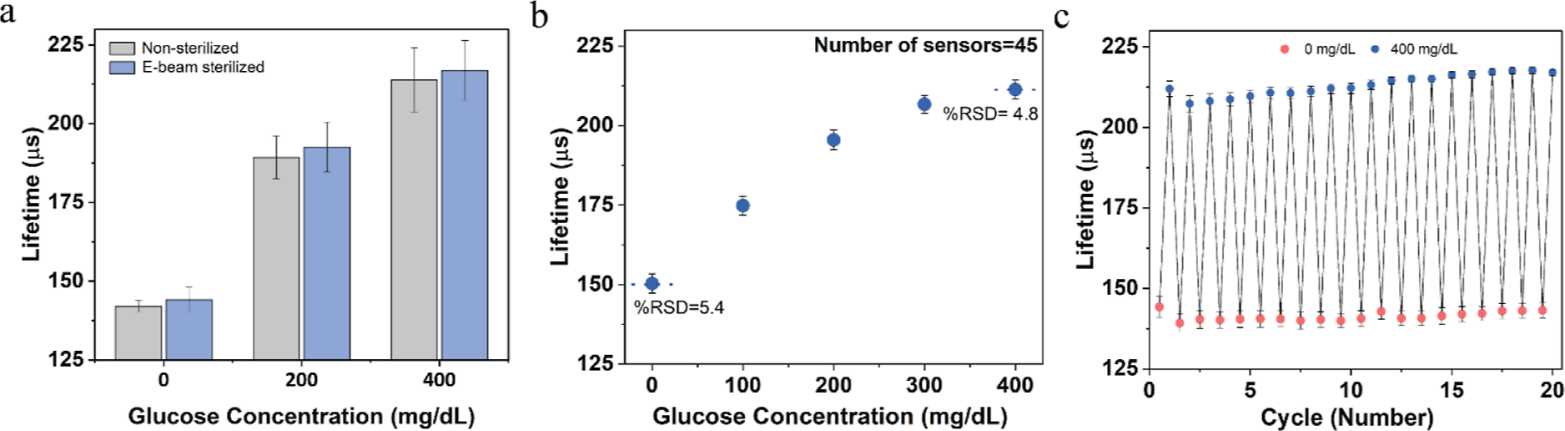
(a) Effect of ∼25 kGy electronic beam (e-beam) sterilization on barcode glucose sensors (*N* = 8). (b) Reproducibility of the barcode glucose sensors (*N* = 45 different sensors) for the detection of glucose ranging from 0 to 400 mg/dL of glucose with relative standard deviation (RSD) value < 6%. (c) Reversibility of barcode sensors (*N* = 4) exposed to 0 and 400 mg/dL of glucose. Error bars represent SD from the mean.

The barcode sensors were subjected to e-beam irradiation in a 10 mM TRIS buffer with a dose of <25 kGy. This sterilization dose of ∼25 kGy has been selected based on ISO 11137-2:2013, which specifies that the minimum e-beam sterilization dose for medical devices typically ranges from 15 kGy to 25 kGy. This dose range is designed to ensure the achievement of the required sterility assurance level of 10^–6^.^40−42^ The lifetime response toward increasing glucose concentration showed only a minor impact, with a lifetime change of ∼6% between nonsterilized and e-beam-sterilized barcode sensors. There were no significant differences (*p* > 0.05) observed at glucose concentrations of 200 and 400 mg/dL, as shown in Figure 6a. Previous studies involving enzymatic glucose and lactate sensors sterilized with the same method exhibited functional sensors but with sensitivity reductions of up to 60% after radiation.^43,44^ Dang et al. observed that when the optical enzymatic sensors were stored in a 5 mM TRIS buffer during radiation, protective effects on the proteins were observed, resulting in an 80% retention of the response when exposed to 15 kGy.^45^ TRIS buffers have radical scavengers that can absorb the free electron energy produced during the e-beam process, thus protecting the enzyme by mitigating the negative effects of e-beam sterilization.^46^ This indicates that immersing the barcode sensors in a TRIS buffer during and after e-beam radiation may have minimized radiation-induced damage.

The sensor-to-sensor reproducibility was also examined by measuring the lifetime responses of 45 replicate barcode sensors consisting of sensing alginate microparticles from two different batches. All the sensors were fabricated under similar conditions and exposed to glucose concentrations ranging from 0 to 400 mg/dL for testing. This yielded an acceptable reproducible response with a RSD of 6% in a lifetime measurement (Figure 6b). To investigate the reversible glucose response, the barcode sensors were exposed to 20 consecutive cycles of 0 and 400 mg/dL glucose concentrations (Figure 6c). The average lifetime response of the sensor at 0 mg/dL glucose concentration was 131 ± 2.6 μs, while the average lifetime response of the sensor at 400 mg/dL glucose concentration was 213 ± 3.2 μs. Together, these findings showed that the barcode sensors can be fabricated to have a very consistent response and with excellent reversibility.

### Storage and Operational/Enzyme Stability

Potential for long-term storage and operational stability is required for the continuous monitoring of metabolites. Over time, enzymatic sensors can degrade due to two factors: enzyme denaturation and hydrogen peroxide poisoning. Hence, both the storage and operational stabilities of the barcode sensors involving the enzymes were evaluated.

**Figure 7:**
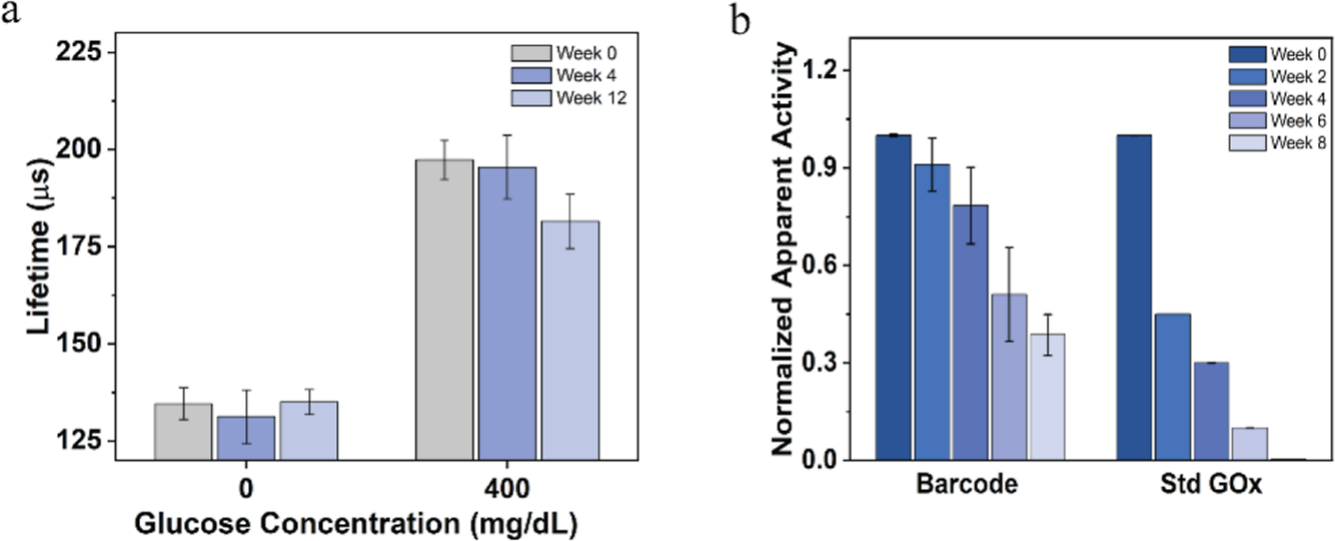
(a) Storage stability of barcode glucose sensors over 12 weeks under storage condition of 4 °C in 10 mM TRIS buffer. (b) Enzyme stability of barcode glucose sensors compared to dissolved GOx over 8 weeks under storage condition of 37 °C in PBS with 100 mg/dL of glucose. Error bars represent the SD from the mean for *N* = 4 different samples.

The lifetime response from barcode sensors stored at 4 °C showed no significant changes after 12 weeks. The sensors retained 94% of their initial response upon exposure to 400 mg/dL of glucose after 12 weeks, as shown in Figure 7a. The relatively high storage stability of the barcode sensors may be due to the immobilization of GOx within the hydrogel microparticles, which has proven to be an effective method of preserving enzyme activity over an extended period.^37,46^ For determining the operational stability of the barcode sensors, both the enzyme activity and the lifetime response were evaluated. Compared to the standard GOx enzyme solution (GOx dissolved in TRIS buffer), which could retain only 3% of the activity by 8 weeks, the barcode sensors were observed to retain 80% of their apparent activity over the first 4 weeks of incubation. However, after 8 weeks of incubation, only 35% of the initial apparent activity was retained (Figure 7b). These findings of enzyme activity loss matched with the observed plateau at <200 μs in the lifetime response at 200 mg/dL of glucose after 5 weeks of incubation, indicating that the system had become enzyme-limited (Figure S5). Previous studies by Moussy’s group have demonstrated that the primary cause of enzyme degradation in glucose sensors is the production of H_2_O_2_.^47^ When glucose sensors are stored without glucose, no glucose oxidation or H_2_O_2_ production occurs, leading to better enzyme stability. In contrast, storage in a glucose solution results in constant H_2_O_2_ production, which gradually degrades the enzyme over time. However, the preserved enzyme stability during operation should be improved by the co-immobilization of CAT, which has been reported to mitigate the effect of hydrogen peroxide produced during glucose catalysis.^48^ Additionally, the incorporation of PAH/PSS nanofilm coating has been reported to sustain the GOx activity encapsulated in alginate microspheres.^37^ Further improvements in sensor longevity could be achieved by loading the sensors with enzymes that have a higher enzyme activity, greater inherent stability, and increased enzyme loading.

### Multianalyte System Response

A crucial aspect of multianalyte detection lies in managing the cross-sensitivity between assays as well as signal crosstalk. Since the phosphorescence lifetime changes from the glucose and oxygen assays are both sensitive to oxygen depletion, the diffusion of oxygen molecules between the neighboring compartments could potentially introduce crosstalk in the barcode sensors if they are positioned too close. For this work, the distance between each sensing compartment was 0.5 mm, which was also used in previous experiments to avoid crosstalk.^23^

To assess crosstalk between compartments, the multianalyte barcode sensors were subjected to cycles of physiologically relevant glucose concentrations (0–200 mg/dL) at a constant oxygen concentration of 40 μM at 37 °C. Between subsequent glucose changes, the barcodes were incubated for 20 min to reach a steady state. The multiplexed lifetime measurements were obtained using the phosphorescence imaging (PLI) reader, which acquired a series of phosphorescence intensity and lifetime images of the entire multianalyte sensor and used these images to derive the lifetime responses of both glucose-sensitive and oxygen-sensitive compartments.

**Figure 8:**
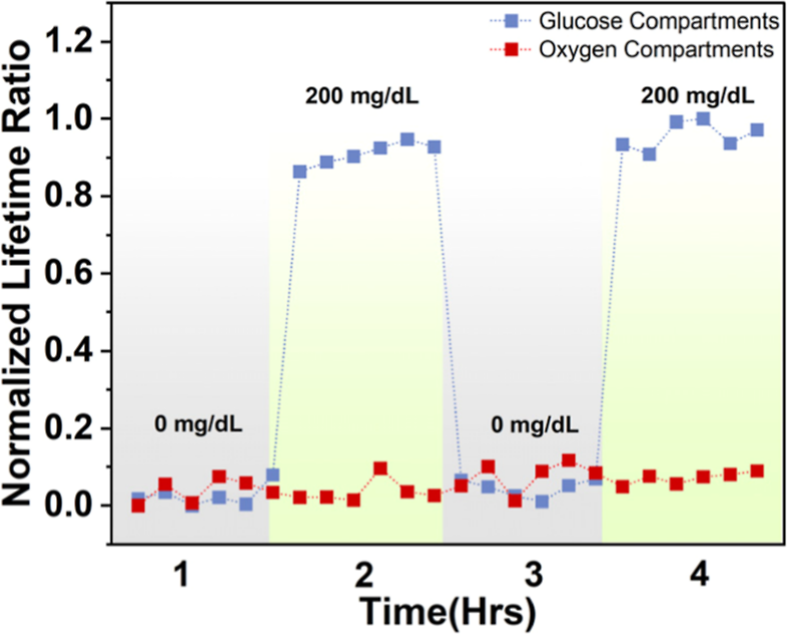
Phosphorescence lifetime (normalized to lifetime at 0 mg/dL glucose concentration) of the barcode hydrogel sensor for glucose- and oxygen-sensitive compartments under two cycles of 0 and 200 mg/dL glucose concentrations.

It was determined that the baseline lifetime in the absence of glucose was comparable for both types of compartments, as expected. Further, the phosphorescence lifetime in glucose-sensitive compartments increased and decreased by 20% when glucose concentrations oscillated between 0 and 200 mg/dL across cycles. Conversely, the glucose-insensitive (oxygen-sensitive) compartments exhibited minimal phosphorescence lifetime variation of only 0.1% in response to the changes in glucose concentration (Figure 8). The slight variations in the oxygen compartments following glucose modulation indicate negligible crosstalk between the sensing compartments.

### In Vivo Studies—Histology

**Figure 9:**
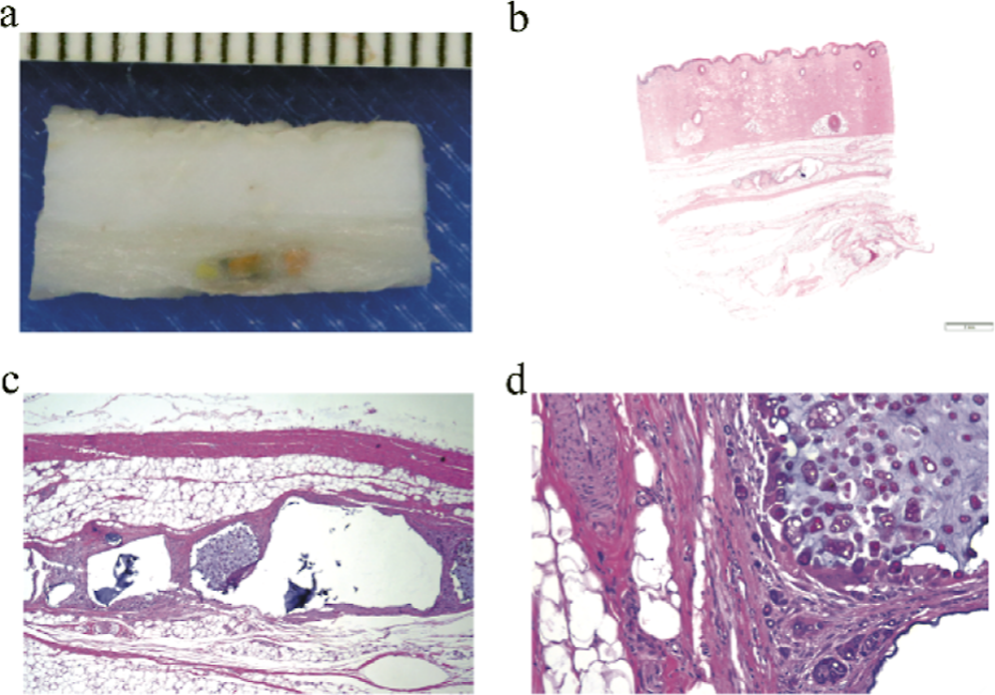
(a) Gross tissue section containing a barcode sensor. (b) Hematoxylin and eosin (H&E) stained subcutaneous tissue section at 5× magnification from porcine study with the barcode sensor. (c,d) Zoomed tissue section, with the sensor demonstrating minimal capsule formation, and tissue healing response.

Histopathological evaluation provides the quantitative data needed to understand the long-term effects of insertable sensors under real-world conditions within the body. For this experiment, independent veterinary pathologists evaluated each sensor location and the surrounding tissue for device presence, device state, skin zone, host interface, and healing response (Figure 9). These sensor evaluations provide insight into the biocompatibility of the barcode sensors.

Pig 1 was evaluated after the barcodes had been inserted for 7 months; (6/9) barcodes were still recoverable, and all barcodes that were located were externally readable and grossly visible on excision. The barcode sensors were mostly recovered from the subcutis (4/6) layers of the skin, with two of the six sensors graded as being in the subcutis/muscle region (2/6). The depths from the surface of the skin to the shallowest portion of each device were also quantified, ranging from 3.84 to 9.19 mm for an average depth of 6.05 mm.

In Pig 2, the barcodes were evaluated after 3 months of subcutaneous insertion. Every barcode could still be detected through the skin, and every barcode was grossly visible in the subcutaneous space after excision. Every barcode was found in the subcutis layer of the skin, with 2/7 barcodes graded as deep dermis/subcutis. The depth from the surface of the skin to the shallowest portion of each device ranged from 2.71 to 5.19 mm, for an average depth of 4.28 mm. The host interface of the barcodes was graded with mild (2/7) to moderate (5/7) fibrous capsule formation and partial (4/7) to diffuse (2/7) infiltrative response from the surrounding tissue. On a single barcode, no infiltration was observed. The body’s healing response was graded as chronic phagocytosis (6/7), with one (1/7) barcode was graded as chronic active phagocytosis. However, a subclinical sarcoptic mite population was detected on histology in Pig 2, which is likely responsible for the eosinophil seen on the slide. This conclusion and attribution of the deleterious response to the presence of mites is supported by the lack of chronic active response to the other sensors in either Pig 1 or Pig 2 and in previous in vivo studies.

In both pigs, the sensors were primarily located in the subcutis. However, implants in the 7 month study in Pig 1 were graded as slightly deeper, erring on the muscle zone rather than the deep dermis zone. They were also somewhat (1.7 mm) deeper in the skin. The different average depths seen in these studies could result from several factors. First, implant positions were randomized, and pigs have widely varied natural skin thicknesses on various parts of their bodies. For example, sensors on the hind flank are naturally under much thinner skin than sensors under the tough shoulder region. Second, because pigs grow rapidly, the pigs doubled in size between the insertion procedures for Pigs 1 and 2, from about 60 pounds to around 120 pounds. This size difference changes the depth of the target subcutis zone. Finally, the sensors may change depth over time as skin thickens and fat deposits thicken as the pig matures. By the end of the experiment, the pigs weighed about 240 pounds each, and the pigs’ skin grew along with the animals. The limited number of animals in this study precludes the easing apart of these several factors. Fortunately, these modest depth variations did not affect the histological findings. More importantly, the ability to read implants at these depths demonstrates the effective penetration of the red excitation light and near-infrared emission through tissue (Figure S6).

In summary, the barcode sensors were well tolerated in healthy animals, remaining readable and visible in the subcutis at about 5 mm under the skin. The typical host response was mild to moderate capsule formation, partial to diffuse cell infiltration, and chronic phagocytosis of the sensor material. Overall, this indicates that the devices are well tolerated over long-term subcutaneous insertion.

## Conclusions

In this study, we demonstrated a highly biocompatible insertable sensing platform for continuous multianalyte sensing achieved by packaging different sensing assays within physically separated compartments within a molded hydrogel. The tunable assays capable of reporting oxygen and glucose at levels expected in tissue were realized by immobilizing oxygen-sensitive phosphors and oxidoreductase enzymes within alginate microparticles coated with polyelectrolyte nanofilms. The polyelectrolyte nanofilms provided the capability to precisely control diffusion into discrete sensing microparticles to balance the diffusion-reaction kinetics at the physiologically relevant low oxygen concentrations. When subjected to cycles of different glucose concentrations, only glucose-sensitive compartments demonstrated changes in the phosphorescence lifetime, and the oxygen-sensitive compartments showed a constant lifetime response, indicating no significant cross-interference between the compartments. Additionally, the barcode sensors demonstrated good stability for long-term storage, reproducibility, the ability to withstand the sterilization process, and excellent biocompatibility. In the future, enzyme stabilization techniques will be exploited to prolong the lifespan of barcode sensors. The addition of other analytes, such as lactate, temperature, and uric acid, will also be explored to broaden the applicability of the barcode sensors to other chronic conditions.

## Supporting Information Available

Schematics of testing systems, phosphorescence lifetime response of oxygen barcodes, key metrics of glucose sensors with different sensing microparticles, raw data illustrating the change in phosphorescence lifetime over time with increases in oxygen concentration and glucose concentration, lifetime response for sensors incubated in glucose solution, and signal-to-noise ratio for barcode sensors implanted in pigs (3 months) (PDF)
