# Supplementary material for: Insertable Biomaterial-Based Multianalyte Barcode Sensor toward Continuous Monitoring of Glucose and Oxygen
Source: ACS Sens. 2024 Nov 4;9(11):6060–70. doi: 10.1021/acssensors.4c01926 (PMC11590103; doi:10.1021/acssensors.4c01926)
Supplement: Supplementary file 1 — se4c01926_si_001.pdf [file se4c01926_si_001.pdf]

Supporting Information

An Insertable Biomaterial-based Multianalyte Barcode Sensor towards Continuous Monitoring of Glucose and Oxygen

Ridhi Pradhan[1], David Chimene[1], Brian S. Ko[1], Artem Goncharov[2,3,4], Aydogan Ozcan[2,3,4,5], Michael J. McShane[1,6*]

*Corresponding Author: mcshane@tamu.edu

[1]Department of Biomedical Engineering, Texas A&M University, College Station, TX 77843 USA.

[2]Electrical & Computer Engineering Department, University of California, Los Angeles, CA 90095 USA.

[3]Bioengineering Department, University of California, Los Angeles, CA 90095 USA.

[4]California NanoSystems Institute (CNSI), University of California, Los Angeles, CA 90095 USA.

[5]Department of Surgery, University of California, Los Angeles, CA 90095 USA.

[6]Department of Materials Science and Engineering, Texas A&M University, College Station, TX 77843 USA.

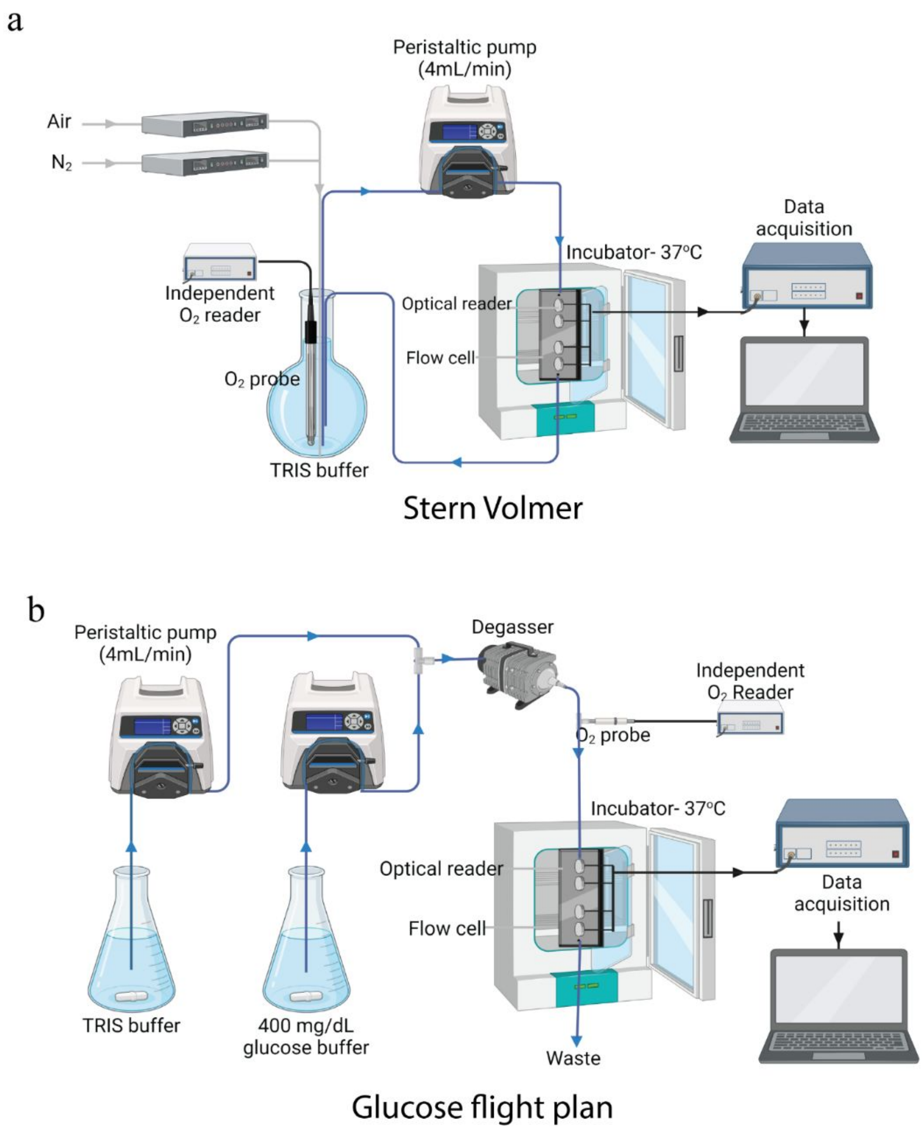

Figure S1: Schematic representation of **(a)**oxygen response testing apparatus, and **(b)** glucose flight plan testing system.

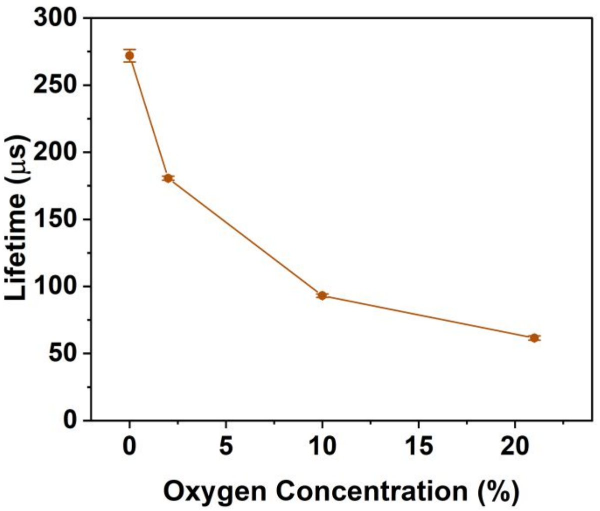

Figure S2: Phosphorescence lifetime response oxygen barcode hydrogel sensor under different oxygen concentrations. Error bars represent the SD from the mean for N=4 different samples.

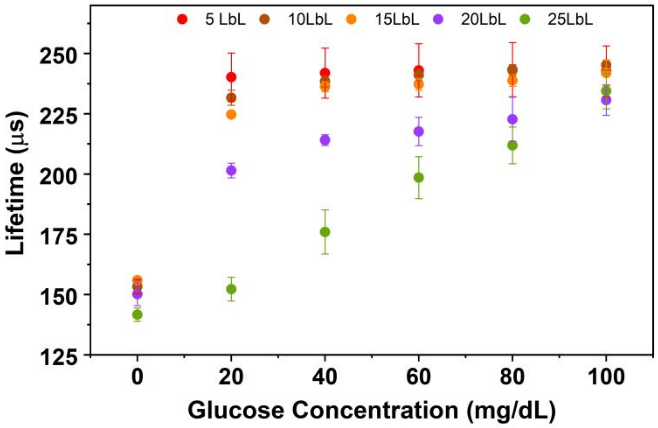

Figure S3: Steady-state lifetime response plots of glucose barcodes (n=4) packed with alginate microparticles with different LbLs, dispersed in barcode hydrogel by challenging the sensors to physiological glucose concentration (0-100 mg/dL) at 40 μM dissolved oxygen concentration and 37°C.

	**LOD[a] (mg/dL)**	**MDGC[b] (mg/dL)**	**Range[c] (mg/dL)**	**Sensitivity (µs-dL/mg)**	**Oxygen Sensitivity (K_{SV}, $\%O_2^{-1}$)[d]**
**Non-crosslinked**					
5 LbL	10.7	65.0	54.3	0.87	0.16
10 LbL	15.3	77.2	61.9	0.72	0.14
15 LbL	14.0	109.0	95.1	0.50	0.14
20 LbL	11.2	112.4	101.2	0.52	0.14
25 LbL	14.6	147.8	133.2	0.37	0.16
**Crosslinked**					
25 CXL	21.3	175.5	154.3	0.38	0.15
30 CXL	37.8	221.3	183.5	0.20	0.15
35 CXL	37.1	321.1	284.0	0.19	0.15

Table S1: Figures of merit calculated for barcode hydrogels with microparticles having uncrosslinked nanofilm coatings (LbL) and crosslinked (CXL) nanofilm coatings comprising different numbers of polyelectrolyte bilayers. Each data represents the average of measurement from 4 different samples. [a]LOD, Limit of detection. [b]MDGC, maximum differentiable glucose concentration. [c]Range, MDGC- LOD. [d]K_{sv}, Stern-Volmer constant.

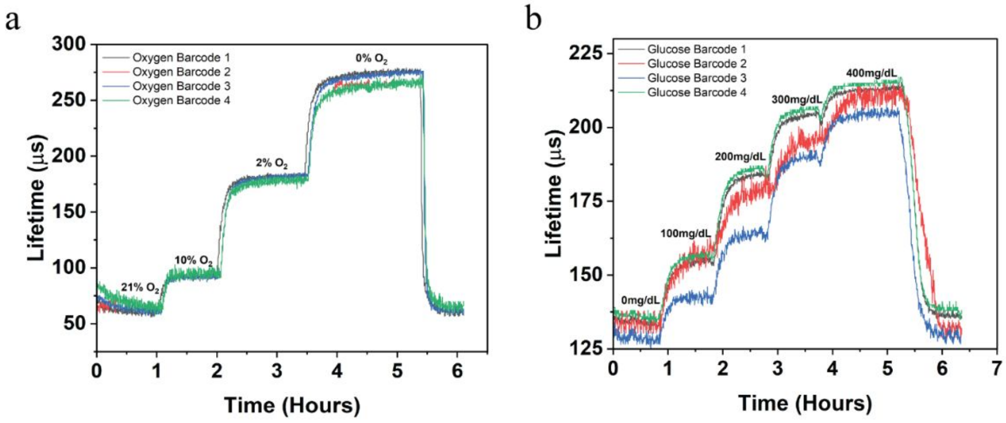

Figure S4: (a) Phosphorescence lifetime response over time to changing dissolved oxygen concentrations for oxygen barcodes. **(b)** Phosphorescence lifetime response over time to changing glucose concentration at fixed oxygen (~30 μM) for glucose barcodes. The average and standard deviations of lifetime measurements were calculated from the plateaus for these data, which were then presented as the steady-state lifetime responses in other graphs (Figures 4, 5, 6, 7a, 8, and S5)

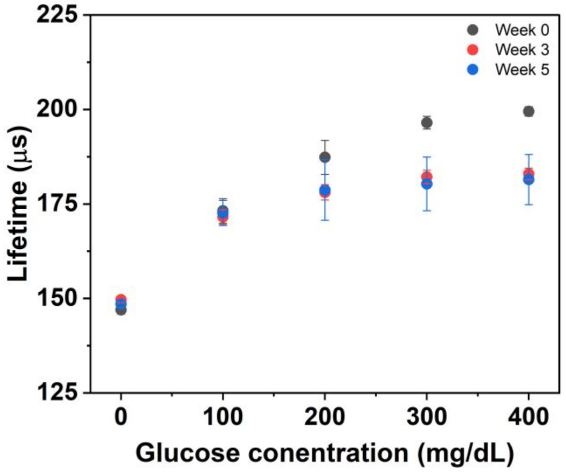

Figure S5: Lifetime response of glucose barcodes stored in PBS (pH 7.4) with 100 mg/dL of glucose at 37°C at time intervals of weeks 0, 3, and 5. Error bars represent the SD of the mean for N=4 different samples.

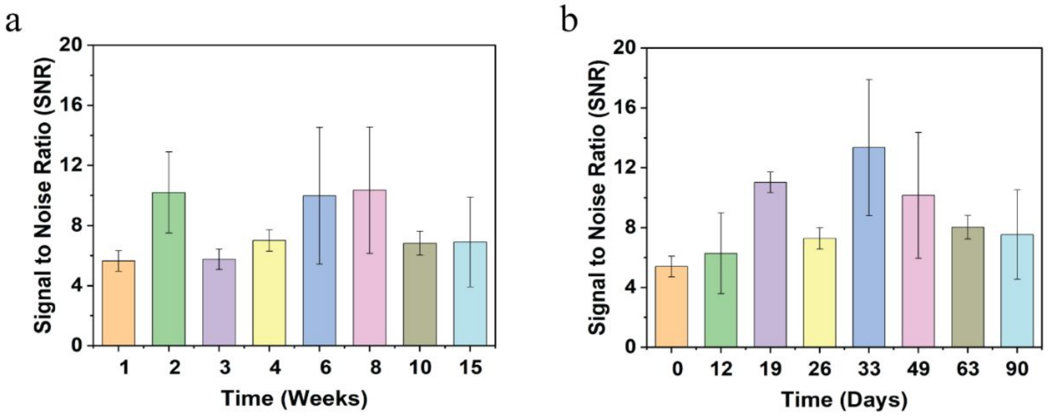

Figure S6: Signal-to-noise ratio (SNR) of barcode sensors implanted in (a) Pig 1 and (b) Pig 2 for a duration of over 3 months. Error bars represent the SD from the mean for N=4 different samples.
